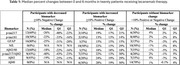# Changes in Alzheimer's disease blood biomarkers in patients undergoing lecanemab therapy

**DOI:** 10.1002/alz70856_099404

**Published:** 2025-12-24

**Authors:** Susan Ashrafzadeh‐Kian, Joshua A Bornhorst, Daniel Figdore, Jonathan Graff Radford, Ronald Petersen, Vijay K. Ramanan, Alicia Algeciras‐Schimnich

**Affiliations:** ^1^ Mayo Clinic, Rochester, MN, USA

## Abstract

**Background:**

Anti‐amyloid therapy trials have reported changes in some Alzheimer's disease (AD) blood biomarkers (BBMs) suggesting that these BBMs may be useful minimally invasive measures of amyloid clearance and treatment outcomes. However, a better understanding of how AD BBMs changes at the individual level reflect treatment response in relation to other relevant clinical endpoints is necessary before they can be adopted for treatment monitoring. Here, several AD BBMs were assessed longitudinally in patients undergoing lecanemab therapy.

**Method:**

Patients undergoing lecanemab therapy at Mayo Clinic, Rochester, MN were recruited to participate in this study. Patients were asked to provide an EDTA‐plasma sample before treatment initiation during the first infusion visit and thereafter at 3‐, 6‐, 12‐, and 18 months post‐treatment. *p*‐tau217, *p*‐tau181, GFAP, NfL, Aβ42, Aβ40, Aβ42/40, and *p*‐tau217/Aβ42 measurements were obtained using Fujirebio Lumipulse assays. Biomarker median differences were assessed between all time points. Patients were grouped into three categories: a decreased biomarker (median decrease of at least 10%), an increased biomarker (median increase of at least 10%), and a stable biomarker (median change < +/−10%.) Biomarker data up to 6 months after treatment is presented.

**Result:**

Of forty‐one patients enrolled since November 2023, twenty had time points up to 6 months. None of the biomarkers showed a statistically significant median change from baseline at 3 months (*p* >0.05). At 6 months, only Aβ42/40 showed a statistically significant median change (decrease) between 0 and 6 months (*n* = 20, *p* = 0.0058). Table 1 shows patients grouped by median biomarker changes from baseline to 6 months. GFAP, *p*‐tau217, and Aβ42/40 exhibited the greatest number of patients with decreased biomarker concentrations (80%, 60%, and 60% of patients with median decreases of ‐25%, ‐26%, and ‐18% respectively), followed by *p*‐tau181 (50% of patients; median decrease ‐22%), *p*‐tau217/Aβ42 (45% of patients; median decrease ‐40%) and Aβ42 (45% of patients; median decrease ‐18%). None of the patients showed a decreased NfL or Aβ40 concentration.

**Conclusion:**

Preliminarily analysis revealed that GFAP, *p*‐tau217, and Aβ42/40 had the most frequent post‐lecanemab biomarker decreases. Additional sample collection is ongoing to assess the association of AD BBM changes with Amyloid PET over time.